# Supplementary material for: Molecular, Cellular and Functional Changes in the Retinas of Young Adult Mice Lacking the Voltage-Gated K+ Channel Subunits Kv8.2 and K2.1
Source: Int J Mol Sci. 2021 May 5;22(9):4877. doi: 10.3390/ijms22094877 (PMC8124447; doi:10.3390/ijms22094877)
Supplement: Supplementary file 1 [file ijms-22-04877-s001.zip › ijms-1060383-supplementary.pdf]

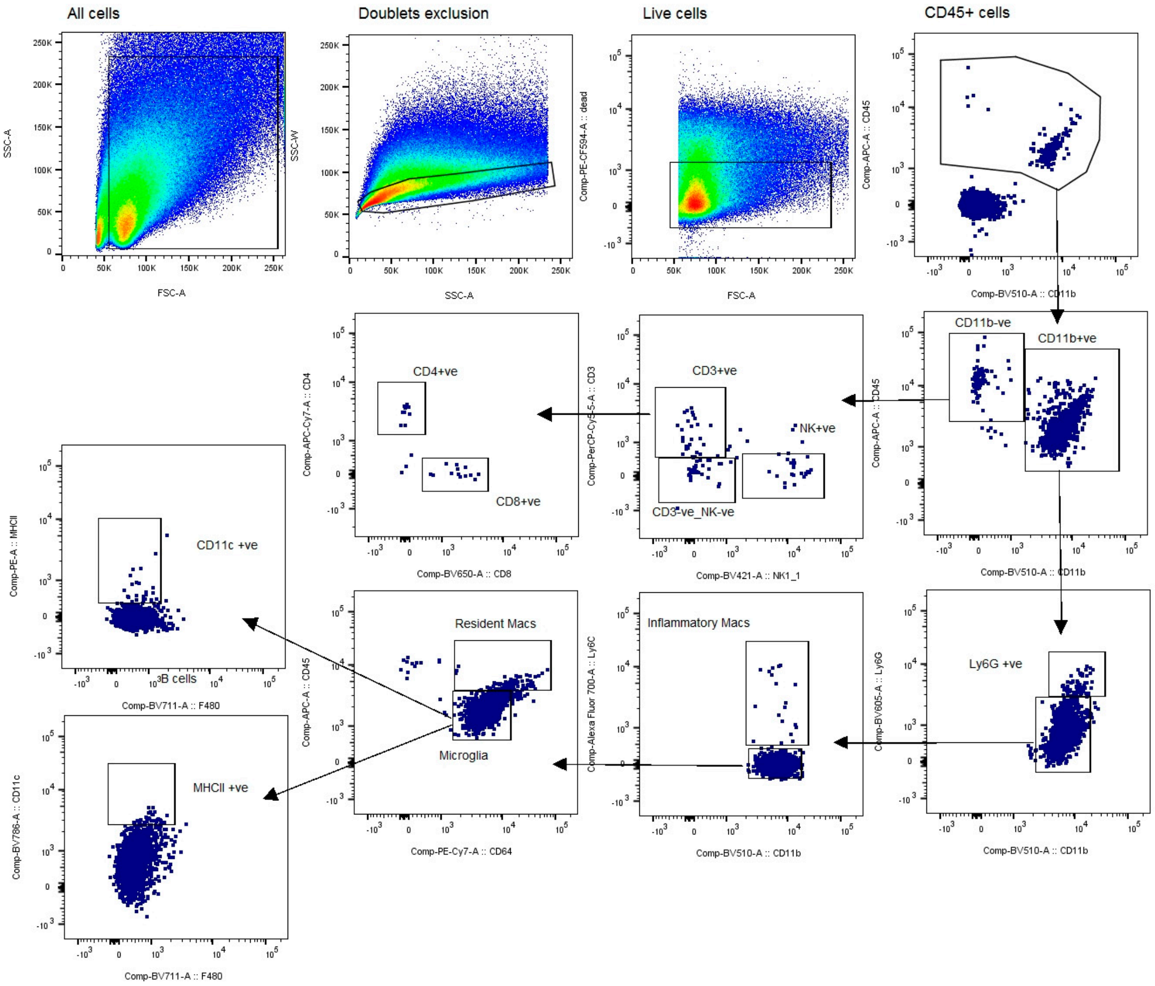

Supplementary Figure S1. Flow cytometry gating strategy. Immune cell populations slocalised to the retina were identified by flow cytometry using the indicated gating strategy.

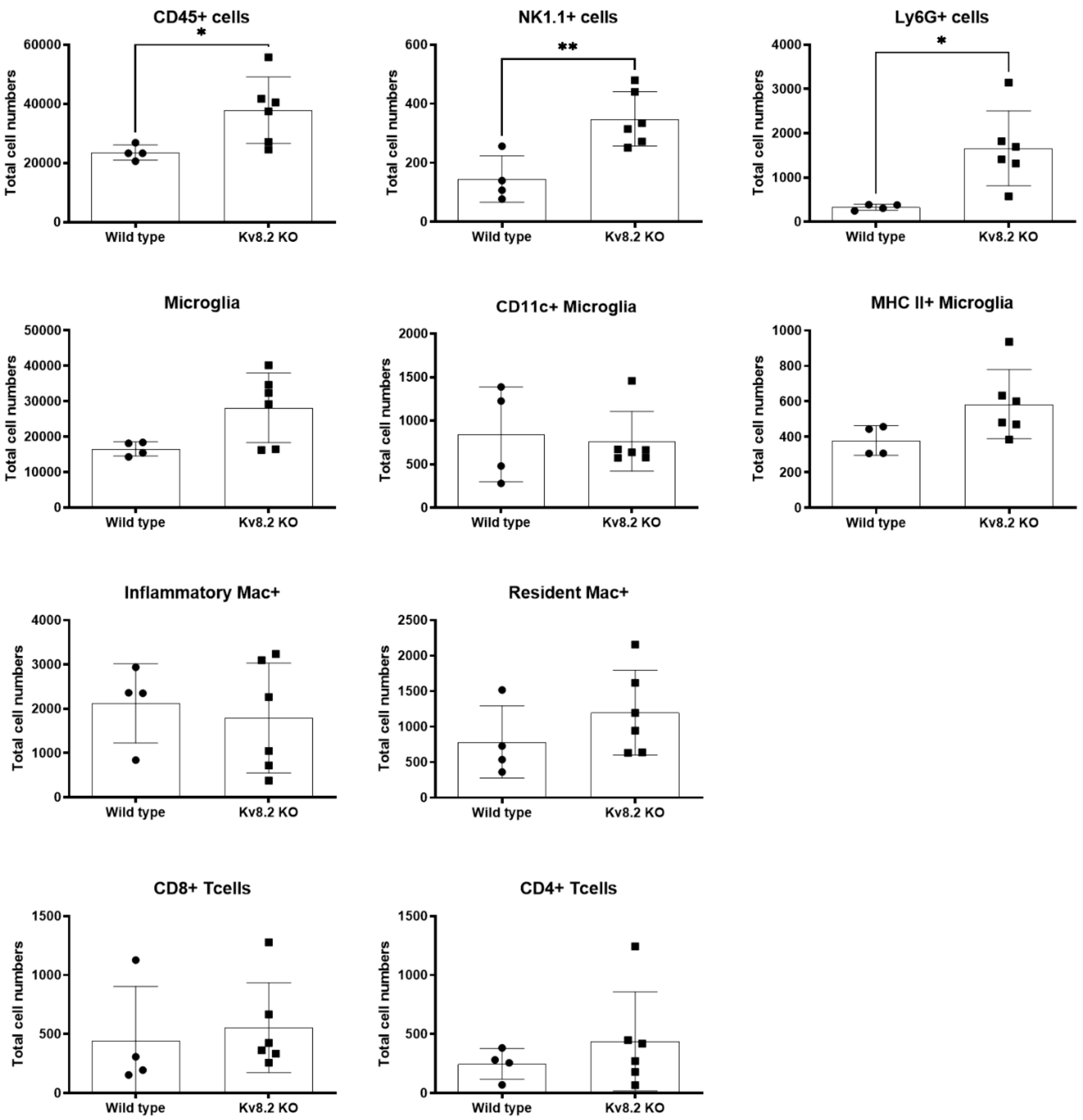

Supplementary Figure S2. Characterisations of immune cells present in the retina of six-month-old WT and Kv8.2 KO mice. Results are presented as mean +/− SEM from n = 6 (Kv8.2 KO) and n = 4 (WT). p values were obtained through unpaired t-test with Welch's correction, * $p < 0.0397$, ** $p = 0.0068$.

Supplementary Table S1. Primary antibody information. IHC, immunohistochemistry.

Antigen	Host Species	Supplier	Cat no.	Working Dilution
Cone Arrestin	Rabbit	Millipore	AB15282	IHC: 1:2000
Kv8.2	Mouse	NeuroMab	73-435	IHC: 1:100
Kv2.1	Mouse	NeuroMab	75-014	IHC: 1:1000
Iba-1	Rabbit	WAKO	019-19741	Flatmounts: 1:500
Rhodopsin	Rabbit	Abcam	AB3424	IHC:1:2000
GFAP	Rabbit	DAKO	Z0334	IHC: 1:500
